# You ≠ me: individual differences in the structure of social cognition

**DOI:** 10.1007/s00426-018-1107-3

**Published:** 2018-10-15

**Authors:** D. J. Shaw, K. Czekóová, C. R. Pennington, A. W. Qureshi, B. Špiláková, M. Salazar, M. Brázdil, T. Urbánek

**Affiliations:** 1 Behavioural and Social Neuroscience Research Group, CEITEC - Central European Institute of Technology, Masaryk University, Kamenice 5, 62500 Brno, Czech Republic; 2 Department of Psychology, School of Life and Health Sciences, Aston University, Birmingham, B4 7ET UK; 3 Institute of Psychology, Academy of Sciences of the Czech Republic, Veveří 97, 60200 Brno, Czech Republic; 4 Department of Health and Social Sciences, Faculty of Health and Applied Sciences, University of the West of England, Coldharbour Lane, Bristol, BS16 1QY UK; 5 Department of Psychology, Edge Hill University, St Helens Road, Ormskirk, Lancashire L39 4QP UK

## Abstract

This study investigated the structure of social cognition, and how it is influenced by personality; specifically, how various socio-cognitive capabilities, and the pattern of inter-relationships and co-dependencies among them differ between divergent personality styles. To measure social cognition, a large non-clinical sample (*n* = 290) undertook an extensive battery of self-report and performance-based measures of visual perspective taking, imitative tendencies, affective empathy, interoceptive accuracy, emotion regulation, and state affectivity. These same individuals then completed the Personality Styles and Disorders Inventory. Latent Profile Analysis revealed two dissociable personality profiles that exhibited contrasting cognitive and affective dispositions, and multivariate analyses indicated further that these profiles differed on measures of social cognition; individuals characterised by a flexible and adaptive personality profile expressed higher action orientation (emotion regulation) compared to those showing more inflexible tendencies, along with better visual perspective taking, superior interoceptive accuracy, less imitative tendencies, and lower personal distress and negativity. These characteristics point towards more efficient self-other distinction, and to higher cognitive control more generally. Moreover, low-level cognitive mechanisms served to mediate other higher level socio-emotional capabilities. Together, these findings elucidate the cognitive and affective underpinnings of individual differences in social behaviour, providing a data-driven model that should guide future research in this area.

**Electronic supplementary material:**

The online version of this article (10.1007/s00426-018-1107-3) contains supplementary material, which is available to authorized users.

## Introduction

We take for granted the sophisticated repertoire of skills required to navigate our way through the myriad of social contexts encountered on a daily basis. Even in simple face-to-face interactions, for example, achieving a desired outcome requires us to infer the mental and emotional states of our interaction partner(s) whilst adapting our own behaviour accordingly. This broad set of cognitive mechanisms is referred to collectively as *social cognition*, and an extensive literature has revealed its highly nuanced and multifaceted composition (see Frith & Frith, 2012; Happé, Cook, & Bird, 2017). Despite this developing understanding, however, an overarching model of social cognition has not yet been specified; it remains unknown if and how its various sub-components are inter-related or co-dependent on one another, or how individual differences in this structure impact on social behaviour.

Research has delineated a variety of socio-cognitive and -emotional mechanisms that enable individuals to interact within social situations (see Frith & Frith, 2012). The conventional perspective on social cognition places each facet within a hierarchical network, in which lower level mechanisms contribute partly towards, or serve as necessary prerequisites for higher level processes (Happé et al., 2017). One such elementary mechanism is* self-other distinction* (SOD), through which we are able to treat independently and distinguish flexibly between representations of the self and others (Lamm, Bukowski, & Silani, 2016; Steinbeis, 2016). Inefficient SOD results in ego-centric misattributions of our own cognitive and affective states onto others, leading us to respond inappropriately in social interactions. This is true, especially when inferring our interaction partners’ perspective or understanding of a situation; since the perspective of others often conflicts with our own, appreciating another’s point of view requires us to detach ourselves from our own viewpoint (Epley, Keysar, van Boven, & Gilovich, 2004). Experimental tasks designed to measure *perspective-taking* attempt to tap into this capacity to resolve self-other conflict, and demonstrate the cognitive cost involved in SOD (Keysar, Barr, Balin, & Brauner, 2000; Samson et al., 2010). Recently, however, conflict resolution has been shown to be just one of two dimensions along which perspective-taking ability varies; compared with individuals reporting an ego-centric attentional focus during social interactions, those reporting more altercentrism or a greater flexibility in attentional focus between self and other exhibit a better perspective-taking performance (Bukowski & Samson, 2016). In this light, variations in attentional focus might underlie the reports of individual differences in perspective taking (e.g., Peterson, Bellows, & Peterson, 2015).

Self-other distinction is also believed to serve a fundamental role in *imitation*. Humans exhibit an involuntary tendency to mimic one another during social interaction, which serves important social functions—it enhances rapport and affiliation among interactants (Chartrand & Lakin, 2013; Leighton, Bird, Orsini, & Hayes, 2010). Neuroscientific investigations have revealed this involuntary behaviour results from a common neural coding (“mirroring”) of self- and other-action (Catmur, Walsh, & Heyes, 2009; Iacoboni et al., 2009). Since our own and others’ actions share a common representational space in the brain, controlling imitative tendencies requires SOD to differentiate between them (Brass, Ruby, & Spengler, 2009; Guzman et al., 2016; Steinbeis, 2016). Consistent with the notion of SOD providing a low-level mechanism common to both perspective taking and imitation, several studies have demonstrated that the expression of involuntary imitation is related inversely to perspective-taking performance (Santiesteban et al., 2012a; b; Shaw, Czekóová, & Porubanová, 2017; Spengler, Bird, & Brass, 2010). Interestingly, attentional focus also appears to exert an influence on imitative tendencies, since individuals scoring high on narcissism show lower involuntary imitation (Hogeveen & Obhi, 2013; Obhi, Hogeveen, Giacomin, & Jordan, 2014).

Neural mirroring mechanisms give rise to brain coupling between actor and observer, thereby affording inter-subjectivity. As such, similar mechanisms might contribute towards *empathy*—that is, an affective state elicited by observing or imagining another person experiencing an emotion, which is both isomorphic to the other’s state yet accompanied by the knowledge that the other person is the source of one’s own affective response (de Vignemont & Singer, 2006). Through neural mirroring, observing another’s emotions might activate our own affective brain systems, thereby allowing us to share in their affective state (Bastiaansen, Thioux, & Keysers, 2009; Lamm, Decety, & Singer, 2011). Recent models distinguish between an affective and cognitive component of empathy (e.g., Shamay-Tsoory, 2011; Shamay-Tsoory, Aharon-Peretz, & Perry, 2009). Given conflicting taxonomies surrounding this dichotomy, we take the former to capture the process of affective sharing (or emotional contagion), and the latter analogous to emotion recognition—a propositional knowledge of the other’s emotional state (see Coll et al., 2017). Since empathic expression is considered a primary trait that drives individual differences in social behaviour (Baron-Cohen, 2009), it is important to elucidate its interdependences on other socio-cognitive and/or -emotional mechanisms that might exert a mediating influence (Kanske, Boeckler, & Singer, 2015). For example, SOD should be necessary to disambiguate between self- and other-affect representations during affective empathy/sharing; inadequate SOD might lead to self-other merging, resulting in personal distress when sharing in others’ negative affective states (Lamm et al., 2016).

Accurate processing and interpretation of affective states elicited during empathy also requires a mechanism of *interoception*, the sensory system through which afferent physiological signals of bodily states are communicated (Tsakiris & Critchley, 2016). Our sensitivity to internal physiological signals influences our subjective experience of emotions and the processing of affective states observed in others (Critchley & Garfinkel, 2017; Critchley, Wiens, Rotshtein, & Dolan, 2004). Indeed, some studies indicate that greater interoceptive accuracy is related to heightened empathic expression (Critchley & Harrison, 2013; Grynberg & Pollatos, 2015). Other studies have found no such relationship, however, suggesting that any link between interoception and empathy is complex, possibly involving other mediating mechanisms (e.g., Ainley, Maister, & Tsakiris, 2015). Those exhibiting high interoceptive accuracy also demonstrate a proficiency in regulating their emotions (Füstös, Gramann, Herbert, & Pollatus, 2012). Conversely, interoceptive hypervigilance is associated with the symptoms of anxiety and depression (Dunn et al., 2010; for a review see Domschke, Stevens, Pfleiderer, & Gerlach, 2010). The direction of this association appears to be mediated by self-referential processing (e.g., self-image; for a review, see Paulus & Stein, 2010). Given its potential influence on other components of social cognition, elucidating individual differences in interoception and its relationship with other aspects of social cognition is central to our understanding of social behaviour (Garfinkel et al., 2015).

Emotional reactions comprise a set of physiological changes that influence our behavioural response to environmental demands; when scared, we might recoil automatically, but, when angered, we might engage aggressively with the provocateur. Although such automatic behavioural reactions can serve important adaptive functions (i.e., fight-or-flight responses), emotion-driven behaviours are often ill-suited to social contexts and must be down-regulated (Gross, 2002). Suppressing impulsive emotion-driven behavioural responses requires cognitive control in the context of emotionally evocative stimuli (Tottenham et al., 2011). *Emotion regulation*, then, is another core component of social behaviour (Schipper & Peterman, 2013). Indeed, emotional dysregulation is observed in clinical syndromes characterised largely by atypical social behaviour, such as autism (Konstantareas & Stewart, 2006), conduct disorder (Davidson, Putnam, & Lawson, 2000), and borderline personality disorder (Fertuck, Lenzenweger, Clarkin, Hoermann, & Stanley, 2006). A number of potential mechanisms appear to underlie this; for example, research demonstrates that the ability to self-regulate emotional states is associated inversely with an individual’s general negative *affectivity* (Eisenberg et al., 1994; Okun, Shepard, & Eisenberg, 2000).

Given the combined influence of attentional focus (alter- versus ego-centrism), interoceptive sensitivity and state affectivity, and the capacity for emotional self-regulation, it follows that any discussion of individual differences in social cognition demands a consideration of *personality*. Very few studies have explored the links between social cognition and personality in the healthy population, however, and those that have report inconsistent findings; while reduced imitative tendencies are reported in narcissism (Hogeveen & Obhi 2013; Obhi et al., 2014), and the self-reported cognitive and affective empathy have been associated with agreeableness and conscientiousness (Melchers et al., 2016), the other studies have found no such relationships (Butler, Ward, & Ramsay, 2015). This contrasts with the findings of investigations into atypical social cognition in clinical samples; social cognitive dysfunction is observed in a number of personality disorders (Herpertz & Bertsch, 2014), and disorder severity is often related to empathic expression (Hengartner et al., 2014). Individuals with narcissistic personality disorder, for instance, exhibit deficits in affective but not cognitive empathy (Herpertz & Bertsch, 2014). Furthermore, patients with borderline personality disorder display impaired emotion regulation and high personal distress when empathising (New et al., 2012), together with poor perspective taking (Semerari et al., 2014; 2015). This may be a consequence of their ego-centric attentional focus (Frick, Lang, & Kotchoubey, 2012). Antisocial personality disorder is also characterised by ego-centrism, coupled with emotional detachment (for a comprehensive review, see Thoma et al., 2013). For this reason, Moroni et al. (2016) have proposed that personality style, rather than diagnostic category, gives rise to these atypical patterns of social cognition.

The sparse research that has investigated relationships between personality and social cognition in the healthy population has employed a variable-centred approach; studies have focused on the degree of relatedness between pre-defined stable personality traits (e.g., neuroticism) and behavioural measures across entire samples (e.g., Butler et al., 2015). The findings from such investigations provide no information on the organisation or dynamics of the mechanisms underlying an individual’s personality, however (Block, 2010; Kuhl, Kazén, & Koole, 2006). An alternative functional approach is offered by Personality System Interaction theory (PSI; Kuhl, 2000a, b), which considers personality to emerge through dynamic interplays between affective dispositions (e.g., sensitivity to negative affect) and the preference for certain modes of cognitive processing (e.g., analytical versus intuitive). In short, there are four sub-systems that interact to structure personality: intention memory (reason), extension memory (self), intuitive behavioural control (habits), and an object recognition system (mistake focus). Interactions between these systems, regulated by positive and negative affectivity, determine an individual’s personality style and, in turn, their behavioural pattern in social contexts. As such, personalities will differ substantially between people who are able to regulate their affective state flexibly according to task demands (action orientation) and those who ruminate in their emotions (state orientation). The PSI framework is conceptualised dimensionally; each dimension is measured on a continuum ranging from personality style to disorder (e.g., charming to histrionic), with disorders proposed to emerge as a result of inflexible (persistent) and maladaptive preferences for a certain style across different situations. This theory may, therefore, be advantageous when investigating individual differences in social cognition. Importantly, PSI theory is informed by neurobiological data (Baumann, Kuhl, & Kazén, 2005; see Kuhl & Quirin, 2011) and supported by behavioural evidence (Jostmann, & Koole, 2007; Kazén, Kuhl, & Quirin, 2015; Koole & Fockenberg, 2011; Quirin, Bode, & Kuhl, 2011). Furthermore, since the functional basis of relationships between affective and cognitive systems is the same for personality styles and disorders, PSI theory permits parallels to be drawn between healthy and clinical populations; it allows us to build upon the recent findings of specific impairments to social cognition in different personality disorders (Moroni et al., 2016; Semerari et al., 2014, 2015) by identifying subgroups of non-clinical individuals with similar profiles of personality style.

To advance our understanding of individual differences in the structure of social cognition, this investigation employed an analytical technique capable of capturing sample heterogeneity by classifying individuals into dissociable subgroups of personality styles defined by PSI. Differences among personality profiles were then explored with a large battery of self-report and performance-based measures employed frequently to assess discrete components of social cognition; namely, perspective taking, imitative tendencies, affective empathy, interoception, and emotion regulation. This allowed us to gain insight into the tapestry of inter-relationships and co-dependencies among these core components of social cognition in the typical population, and the potentially differentiating influence of personality.

## Methods

### Participants

Three hundred and seven right-handed individuals (122 males) from Brno, Czech Republic, participated in this study, but data from four of these individuals were omitted, because they represented multivariate outliers. Data from the remaining 303 individuals (122 males; *M*_age_ = 23.1 years, SE = 0.18) were used for personality assessment, with all individuals reporting normal or correct-to-normal eyesight and no neurological or psychiatric diagnoses. After excluding from this initial personality sample any individuals from whom we had incomplete data across all measures of social cognition (*n* = 13; see “Statistical Analyses”), further analyses were conducted on a sub-set of 290 individuals (117 males; *M*_age_ = 23.03 years, SE = 0.19). Sensitivity power analyses indicated that this sample size was sufficient to detect the smallest effect size of interest (*r* > .10) with 80% power, consistent with guidelines on individual differences (Gignac & Szodari, 2016). Comprehensive participant demographics are provided in Supplementary Table S1 (see *Supplementary Material*). The study was approved by the Ethics Board of the Institute of Psychology, Academy of Sciences in the Czech Republic. All individuals provided informed consent prior to their participation in the study, and were recompensed with 200 Kč (approx. €7.5).

## Measures and procedure

Participants completed a personality assessment prior to their arrival in the laboratory. Individual facets of social cognition were then measured using a variety of computerised measures administered in Cogent (v1.31; http://www.vislab.ucl.ac.uk/cogent), a toolbox for MATLAB (vR2015b; The MathWorks Inc., Natick, MA).

### Personality

Personality was assessed with the Personality Styles and Disorders Inventory (PSDI; Kuhl & Kazén, 2002), a self-report instrument borne out of PSI theory. The PSDI comprises 140 items, each rated on a four-point Likert scale (0 = “*Certainly no*”, 3 = “*Certainly yes*”). Together, these items measure 14 personality dimensions, which are presented in Supplementary Table S2 along with reliability measures (all *a* > 0.64). Importantly, personality styles identified with this instrument differentiate on a variety of cognitive tasks (e.g., Urbánek, & Marček, 2016).

### Imitative tendencies

Imitation was assessed with a stimulus–response compatibility task (Brass, Bekkering, & Prinz, 2001), whereby participants are required to execute finger-lifting actions in response to a coloured dot (imperative stimulus) while observing task-irrelevant finger actions performed by a model’s hand (stimulus hand). The degree to which participants are faster and more accurate at executing finger movements signalled by the imperative stimulus when they observe simultaneous compatible (matching) compared with incompatible (opposing) movements is referred to as automatic imitation, and is considered an experimental measure of spontaneous mimicry. Genschow et al. (2017) reported a high split-half reliability of 0.86 for this compatibility effect.

To avoid the confounding influences of anatomical, spatial- and orthogonal-compatibility effects (Shaw et al., 2017), the observed finger actions were performed by a left or right stimulus hand rotated, respectively, clockwise (+ 90°) or counter-clockwise (− 90°) from the participants’ perspective (see Supplementary Figure S1). Each trial began with the stimulus hand resting on a flat surface, signalling that participants should depress both the left and right directional arrows on a standard keyboard with the index and middle finger of their right hand, respectively. After 800, 1600, or 2400 ms, selected randomly, the warning stimulus was replaced with an end-point image of the same stimulus hand performing either an index or middle finger extension. In this end-point image, a dot was presented between the index and middle finger, the colour of which served as the imperative stimulus—it signalled whether the participant should extend their own index or middle finger (the colour-finger pairing was counterbalanced across participants). In response to the imperative stimulus, participants lifted the corresponding finger as quickly as possible, thereby releasing a key. The trial then ended with a blank screen presented for 1000 ms. The combination of imperative stimulus and stimulus hand resulted in four trial types: compatible (the same finger action was both signalled and observed), incompatible (opposite finger actions were signalled and observed), baseline (a movement was signalled, but the stimulus hand did not move a finger), and catch (no movement was signalled, but the stimulus hand moved a finger). The procedure comprised two blocks of 90 trials—30 compatible, 30 incompatible, 20 baseline, and 10 catch—with accuracy and response time (RT) measured on each trial. Each block presented either the + 90° or − 90° rotation of the stimulus hand, with the order of blocks counterbalanced across participants. Five practice trials were completed before the task began. To be consistent with the vast majority of stimulus–response paradigms employed to investigate automatic imitation and its relationship with other socio-cognitive variables (Cracco et al., 2018), we subtracted mean RT on the incompatible from the compatible condition. We refer to this herein as automatic imitation, with higher scores representing greater imitative tendencies.

### Visual perspective taking

The director task (e.g., Keysar et al., 2000) was employed to measure low-level (level-one) visual perspective-taking (VPT). This task requires participants to move objects around a grid of shelves according to verbal instructions given by a ‘Director’. Sometimes, the grid display affords two competing perspectives; the participants’ viewpoint from the front of the display differs from the Director’s vantage point from the rear. Under these conditions, the participants must ignore their own self-perspective and act according to their inference of the Director’s perspective.

An example trial from each of the three conditions is illustrated in Supplementary Figure S1. On each trial, the stimulus consisted of an 8 × 8 grid of shelves forming 16 boxes, five of which had opaque backs. Various objects were placed within eight of these boxes, and the participant was presented with a recorded verbal instruction from the Director to move one of the objects to a different box. The Director was located behind the shelf display in all the conditions, which meant that she was unable to see the contents of the five opaque boxes—their contents were visible only from the participant’s (front) perspective. On experimental trials, the Director’s instruction referred to an object in one of the opaque boxes; in the illustrated example, the Director would instruct the participant to “move the smallest apple”. This created a discrepancy between the Director and participants’ perspectives, and to follow the instructions correctly on these trials, the participant had to discount any “distractor” objects not visible to the Director (e.g., they were to move the medium-sized apple rather than the smallest). In the first- and second-control conditions, there was no distractor object to discount; it was replaced in the first, and in the second, the Director’s instruction referred to an object in a box that they could see. Each condition comprised 20 trials presented pseudo-randomly. The recordings of verbal instructions were equivalent across all 60 trials [mean duration = 3.26 (SD = 0.22) s]. Participants responded by indicating with the mouse into which box the object should be moved, and accuracy and RT were measured on each trial.

In line with our previous research (Shaw et al., 2017), in which we revealed a relationship between VPT and automatic imitation using the exact same experimental procedures, we used a residualised measure of difference between the conditions: First, at the participant level, to account for any speed-accuracy trade-off, we calculated inverted efficiency scores in each condition by dividing the mean RT by the proportion of correct responses. This was justified, since RT and accuracy were correlated in all conditions (*r* > 0.12, *p* < 0.043; see Bruyer & Brysbaert, 2011). We then averaged inverted efficiency scores over the two control conditions. Finally, across the entire sample, we regressed these averaged scores across the control conditions from those in the experimental condition. This produced standardised residuals for each participant, with higher values representing poorer performance on trials, whereby their own perspective differed from the director’s relative to those in which no such discrepancy existed. To our knowledge, there has been no formal assessment of reliability for the director task, so we assessed the split-half reliability of our data from this performance measure. Both RTs and accuracy demonstrated excellent split-half reliability in each condition (> 0.96).

### Interoception

We assessed interoception implicitly by measuring heartbeat estimation with the mental tracking method (Schandry, 1981)—a well-validated technique with the reports of test–retest reliability up to 0.81 (Knoll & Hodapp, 1992; Werner et al., 2013). This task measures the accuracy with which individuals count their own heartbeat, a measure that correlates highly with awareness of other bodily cues (e.g., gastric events; Herbert et al., 2012).

To ensure accurate measurements of heart rate, participants were instructed not to drink caffeinated drinks or smoke cigarettes 1 h prior to the experimental procedure. Prior to the task, participants were instructed to rest for 5 min, allowing their heart rate to return to resting levels. Participants then completed a heartbeat estimation task, which comprised a four block sequence of different time intervals (25, 35, 45, and 55 s) presented in a pseudo-random fashion, with inter-block intervals ranging between 10 and 30 s. Throughout all the blocks, participants wore noise-cancelling headphones through which white noise was played and were asked to close their eyes. Participants received the following the standardised instruction: ‘*Without checking, can you count silently each heartbeat you feel in your body from the first to the second tone. Any kind of physical manipulation, such as holding your breath or evaluating your pulse, is not allowed*’. After each block, the participant was asked to state verbally the number of heartbeats that they had counted in that interval.

Actual heartbeat was measured during each interval via the electrocardiogram (ECG), recorded with a PsychLab EEG8 amplifier unit (http://www.psychlab.com/EEG_8_amplifier.html) sampling at 1000 Hz and Ag-AgCl surface electrodes positioned on the participant’s left and right wrist. The ECG data acquired during the task were analysed offline using a processing pipeline in MATLAB (http://www.librow.com/cases/case-2), which involves a Fourier transformation to remove low-frequency drifts from the recording and the subsequent identification of local maxima considered to be R-peaks. Our measure of interoceptive accuracy was calculated according to the formula given by Garfinkel et al. (2015):

$${\text{1 }} - {\text{ }}\left( {|{\text{nbeat}}{{\text{s}}_{{\text{real}}}} - {\text{ nbeat}}{{\text{s}}_{{\text{reported}}}}|} \right)/\left( {\left( {{\text{nbeat}}{{\text{s}}_{{\text{real}}}}+{\text{nbeat}}{{\text{s}}_{{\text{reported}}}}} \right)/{\text{2}}} \right).$$

### Emotion regulation

#### Implicit measure

Emotion regulation was measured implicitly with an emotional Go/No-Go task, which assesses individuals’ ability to maintain cognitive control over impulsive responses to emotionally evocative stimuli (e.g., Tottenham et al., 2011). The previous research using the Go/No-Go task reports good test–retest reliability (0.65; Weafer, Baggott, & de Wit, 2013).

This task comprised six blocks of 40 trials. In each block, a sequence of face stimuli was presented in rapid succession, each face showing one of two expressions. At the beginning of each block, participants were instructed to press the space bar as quickly as possible whenever a particular expression was presented. These “Go” trials occurred frequently (70%; 28) in a given block to instil a pre-potent tendency for the participant to respond. The remaining “No-Go” trials (30%; 12) presented participants with a different emotional expression. Participants were instructed to withhold the response for any facial expression other than the “Go” expression. The task comprised three emotional (angry, fearful, and happy expressions) and three non-emotional blocks (neutral expressions). In any given block, an emotional expression was always paired with a neutral expression; the emotional expression served as either the “Go” stimulus (with the neutral expression as the “No-Go” stimulus) or the “No-Go” stimulus (with the neutral expression serving as the “Go” stimulus). The blocks comprising these pairings were performed in counterbalanced order: Angry–neutral and neutral–angry; fearful–neutral and neutral–fearful; happy–neutral and neutral–happy. Each trial started with a fixation cross presented for 1000–2000 ms (*M* = 1500), followed by the face stimulus presented for 500 ms (see Supplementary Figure S1). The order of Go and No-Go trials was pseudo-randomised to ensure that no two No-Go trials occurred in succession. Prior to the task, participants performed a short practice block with a different stimulus set to that used in the experimental blocks. The face stimuli were selected from the Radboud Faces Database (14 males; Langner et al., 2010); each one grey scaled and cropped to remove any hair.

As a measure of emotion regulation, we extracted the proportion of trials in which the participant incorrectly responded to an emotional No-Go stimulus; that is, the false-alarm rate on emotional blocks (Tottenham et al., 2011). Following the approach taken by Tottenham et al. (2011), these values were z-scored by normalising to the sample standard deviation. Importantly, higher values on this measure represent poorer emotion regulation ability.

#### Explicit measure

To assess emotion regulation via self-report, participants completed the Action Control Scale (Kuhl, 1994), an instrument that measures an individual’s ability to regulate affective states quickly and flexibly in response to environmental demands (action orientation) rather than fixating on them in a change-preventing volitional mode that allows them to impact upon behaviour (state orientation).

This 36-item questionnaire consists of three sub-scales, each measured by 12 items: action orientation after failure versus preoccupation (AOF); demand-related action orientation versus hesitation (AOD); and action orientation during activity performance versus volatility (AOP). Each item presents an everyday situation (e.g., “*When I am told that my work has been completely unsatisfactory*”), and participants select one of two possibilities—one indicative of action orientation (“*I don’t let it bother me for too long*”) and the other of state orientation (“*I feel paralyzed*”). Since AOP is considered less relevant than AOF and AOD to the personality theory (see below; Kuhl, 1994), we focus only on the latter two dimensions herein. These two sub-scales achieved acceptable reliability (*a* > 0.74; see Supplementary Table S2).

### Empathy

#### Implicit measure

To measure empathy implicitly, we developed a task that followed the same principles as the Multifaceted Empathy Test (Dziobek et al., 2008); a performance measure proven effective in dissociating between the cognitive and affective components of empathy at the behavioural and neurophysiological level, and in healthy and clinical samples (Mazza et al., 2014; Moore et al., 2015).

In our adaptation (see Czekóová et al., 2016), participants were presented with 30 colour photographs of individuals experiencing different emotions in various contexts. The task involves two blocks, with each photograph presented randomly in each block. In the first block, participants were required to select one of four options that best describes how they believe the person in the image is feeling (cognitive empathy). In the second block, they rated their own arousal in response to observing the emotion expressed by the person in the image (affective empathy) on a 7-point Likert scale (1 = “*None*”, 7 = “*Very strong*”; affective empathy). Both accuracy and arousal ratings were recorded. In both blocks, each image was presented for a maximum of 10 s. Internal consistency was excellent for measures of affective empathy (Cronbach’s *α* = 0.84). Unlike our initial piloting, however, in which we achieved good levels of reliability for the measure of cognitive empathy (*n* = 112, *α* = 0.67), this measure achieved unacceptable reliability in the present sample (*α* = 0.47). For this reason, we focus only on the former measurement herein, referred to henceforth as affective empathy.

#### Explicit measure

We also obtained an explicit, self-report measure of empathy with the Interpersonal Reactivity Index (Davis, 1983), a multidimensional measure of individual differences in trait empathy shown to be valid and highly reliable across a range of populations pertinent to the present sample (mid- to late-adolescence [Hawk et al., 2013], various European countries [De Corte et al., 2007; Gilet et al., 2013], and experimental hypotheses [autistic individuals; Rogers et al., 2007]).

This 28-item instrument consists of four seven-item sub-scales that measure discrete empathic tendencies: Perspective taking (adopting spontaneously the psychological perspective of others), Fantasy (transposing oneself imaginatively into the feelings and actions of fictitious characters), Empathic concern (adopting “other-oriented” feelings of sympathy and concern), and Personal distress (having “self-oriented” feelings of personal anxiety and unease in tense interpersonal settings). The Perspective taking and Fantasy sub-scales’ index cognitive empathy, whilst Empathic concern and Personal distress reflect affective empathy. Participants indicated their answer for each item on a five-point Likert scale (1 = “*Does not describe me well*”, 5 = “*Describes me very well*”). Given that we were interested primarily in empathy as it unfolds during real-world social interactions, we focused only on perspective taking, empathic concern, and personal distress (for related discussions see De Corte et al., 2007; Su, Lee, Ding, & Comer, 2005) [see Supplementary Table S2 for the internal consistency of all three sub-scales (*α* > 0.73)].

### State affectivity

To measure participants’ state affectivity at the time of testing, we employed the Implicit Positive and Negative Affect Test (Quirin, Kazén, & Kuhl, 2009)—a task designed to measure implicitly an individual’s positive and negative affective state. This measure has been shown to have good test–retest reliability (> 0.72) and construct validity (Quirin et al., 2009; van der Ploeg et al., 2016), and to predict physiological indices of affectivity (e.g., cortisol levels) much better than direct self-report instruments (Quirin & Bode, 2014).

The test consisted of six artificial words (e.g., “SAFME” and “TALEP”) that participants were asked to rate in terms of the extent to which they conveyed six different mood states (happy, cheerful, energetic, helpless, tense, or inhibited), using a four-point Likert scale (1 = “*Does not fit at all*”, 4 = “*Fits very well*”). In a pseudo-random order, each non-word was presented six times alongside one of the six mood-state adjectives, resulting in 36 trials. Ratings across both positive and negative adjectives showed acceptable internal consistency (Cronbach’s *α* = 0.71 and 0.66, respectively), and positive and negative affectivity scores were uncorrelated (*r*_(288)_ = 0.08). Given its known relationship with the other measures of social cognition (Eisenberg et al., 1994; Okun et al., 2000), we focused our analyses only on scores of negative affectivity (referred to herein as negativity).

## Statistical analyses

After the removal of four multivariate outliers across tasks, Latent Profile Analysis (LPA) was performed on the personality data from the initial sample of 303 individuals to identify distinct profiles of personality styles. Due to technical or performance failures, however, some of these behavioural measures were missing or incomplete for 13 participants (5 males). Since we were interested in data from individuals who performed all measures, Multivariate Analysis of Variance (MANOVA) and Structural Equation Modelling (SEM) were performed on a final sample of 290 participants to compare personality profiles across each measure of social cognition, and the inter-relationships among them. Where measures violated the assumption of normality, non-parametric analyses were conducted. Values below present means (± SE).

### Latent Profile Analysis

Latent Profile Analysis (Lazarsfeld & Henry, 1968) was conducted on PSDI data to classify participants into homogeneous sub-groups on the basis of preferred personality styles across all personality dimensions. LPA is a data-driven, model-based analytical technique that uncovers hidden groups within a sample of individuals, while taking into account uncertainty of group membership for each participant. Models with one to five latent profiles were calculated to identify the best-fitting model. The optimal model solution was determined on the basis of several fit indices, including Akaike Information Criteria (AIC; Akaike, 1974), Bayesian Information Criteria (BIC; Schwarz, 1978), sample-size adjusted BIC, the Lo-Mendell-Rubin Adjusted Likelihood Ratio Test (LMRT; Lo, Mendell, & Rubin, 2001), the Parametric Bootstrapped Likelihood Ratio Test (BLRT; McLachlan & Peel, 2000), and entropy, as well as with respect to the previous research. The LMRT and BLRT reflect a significant improvement in model fit after comparing a given profile solution with a model that contains one less profile. Entropy summarises the degree of accuracy in classifying participants into discrete profiles on the basis of posterior probabilities, with higher values signifying a better profile separation (Ramaswamy, DeSarbo, Reibstein, & Robinson, 1993). While there is no general consensus on which fit indices should be preferred in case of their disagreement, the past research suggests that some indicators tend to perform better than others—BIC and sample-size adjusted BIC, for instance, have been shown to be more accurate in identification of correct solution compared to AIC, which tends to overestimate the number of profiles. Furthermore, the BLRT has been found to outperform LMRT as well as the rest of above-listed indices across various models and sample sizes (Nylund, Asparouhov & Muthén, 2007; Morgan, 2015). For this reason, we used maximal convergence among all fit indices to determine the optimal number of profiles.

The analyses were conducted in Mplus 7.3 (Muthén & Muthén, 2012), with 100 bootstrap samples drawn from all the models for BLRT.

### Performance measure checks

The stimulus–response compatibility task successfully elicited automatic imitation; participants were faster to respond correctly to the imperative stimulus on compatible compared with incompatible trials [480.12 (± 3.71) vs. 487.96 (3.82) ms; Z_(290)_ = 3.27, *p* = 0.001]. Accuracy was equivalent between the conditions; however, [94.79 (± 0.33) vs. 94.81 (± 0.34) ms; Z_(290)_ = 0.48, *p* = 0.630].

When measuring VPT, participants were expectedly slower and less accurate on the experimental condition [4.98 (± 0.07) s, 81.28 (± 1.04) %] compared with control conditions one [4.94 (± 0.06) s, Z = 2.29, *p* = 0.022; 87.93 (± 0.95) %, Z = 9.57, *p* < 0.001] and two of the Director Task [4.88 (± 0.05) s, Z = 4.52, *p* < 0.001; 86.53 (± 0.92) %, Z = 7.76, *p* < 0.001].

### Structural equation modelling

We conducted SEM to investigate directional relationships between our various measures of socio-cognitive and -emotional abilities, and the influence of personality. First, all pairwise plots were examined for nonlinearity and heteroscedasticity, and the distributions of the dependent variables were checked for multivariate outliers (c.f. Tabachnik & Fidell, 2013) before the variance of each variable was examined and modified as per Kline (2015). We then defined a default model—a reference against which any subsequent modifications suggested by the SEM analysis could be evaluated (e.g., addition or removal of directional paths and covariances). We employed a two-step process to arrive at a default structure objectively: first, we performed an MANOVA to identify variables that did and did not differ* directly* between the two personality classes. Having identified variables that did not differ, we then entered each into a mediation analysis to examine whether or not they differed between personality profiles *indirectly* via the influence of other measures. This approach follows contemporary recommendations that argue against the first condition of the causal steps approach (Baron & Kenny, 1986); specifically, the causal and outcome variable do not need to be correlated for the identification of important mediating influences (Hayes, 2013; Kenny & Judd, 2014; Zhao, Lynch, & Chen, 2010). Only variables that differed significantly between the personality profiles and correlated with those that did not differ were considered as mediators, however, thereby satisfying the second and third conditions of the causal steps approach. Mediation analyses were conducted using ordinary least-squares path analysis (Hayes, 2013). Indirect effects were assessed with 1000 bias-corrected bootstrap confidence intervals to ensure that, for significant paths, these did not overlap zero.

Having defined a default model by combining all fully or partially mediated models, in a step-by-step manner, we implemented structural changes suggested by modification indices that decreased disparity in fit between the observed and modelled data; specifically, changes that resulted in significant decreases of *χ*^2^, AIC, and the BIC.

## Results

**Fig. 1:**
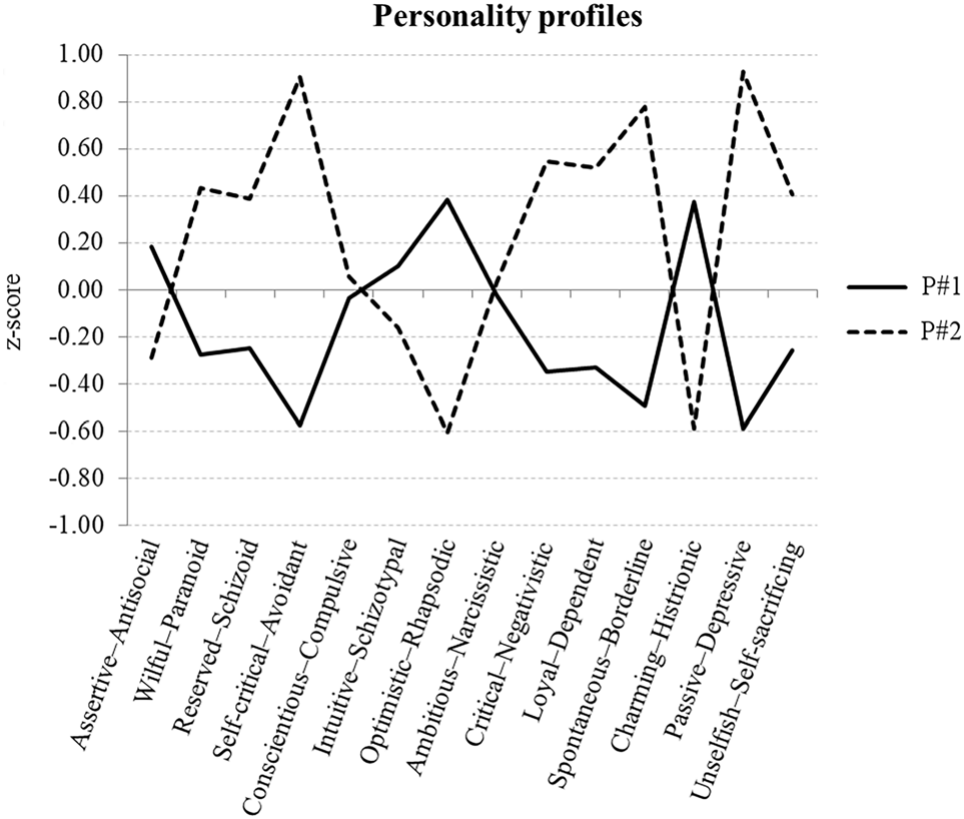
Profiles emerging from Latent Profile Analysis. Negative scores suggest a style is used infrequently, whilst positive scores indicate a strong preference (inflexibility) for that style

All fit indices from the LPA suggested that a two-profile solution was optimal (see Supplementary Table S3). Figure 1 illustrates these two profiles of personality styles, with negative scores, indicating that a style is used infrequently and positive scores suggestive of a tendency (persistence) for that style. Latent profile 1 (P#1) comprised 187 participants (61.7%; 78 males), and is characterised by low scores on the Self-critical-Avoidant, Spontaneous-Borderline, and Passive-Depressive styles. Instead, individuals in this profile demonstrate a slight tendency for the Optimistic–Rhapsodic and Charming–Histrionic styles. Importantly, however, these style preferences are less strong than those shown by profile 2 (P#2), indicating less rigidity (greater flexibility). The 116 participants (38.3%; 44 males) comprising P#2 scored low on Optimistic–Rhapsodic and Charming–Histrionic personality styles but especially high on the Self-critical–Avoidant, Spontaneous–Borderline, and Passive–Depressive styles. Such strong scores on these personality styles indicate relative inflexibility. No significant differences between the profiles were found with respect to demographics such as sex, age, marital status, or completed education (see Supplementary Table S1). To check the reliability of the participant classification and profile characterisation, we performed two split-half validations; the sample was divided randomly into halves and a two-profile LPA was performed on each of the four sub-samples. Aside from a single instance of misclassification in one sub-sample, profile classifications were equivalent across all four iterations, as was the characterisation of the two profiles (see Supplementary Figure S2).

As shown in Table 1, an MANOVA revealed that mean scores on seven of our 11 measures of social cognition differed significantly between the two personality profiles. In line with the results of the LPA, individuals showing greater flexibility between styles (P#1) scored higher on both dimensions of action orientation (AOF and AOD), and lower on Personal distress and negativity relative to P#2. These individuals also reported higher Perspective taking, which showed superior interoceptive accuracy and exhibited considerably lower automatic imitation compared with P#2.

**Table 1:** Direct group comparisons

Measure	Profile means		*F*	*p* value	*η*_p_^2^
	#1	#2			
AOF	6.22 (± 0.20)	3.51 (± 0.26)	70.44	< 0.001	0.20
AOD	6.69 (± 0.20)	4.15 (± 0.30)	62.16	< 0.001	0.18
PT	18.82 (± 0.33)	17.60 (± 0.43)	5.05	0.025	0.02
EC	18.85 (± 0.37)	19.60 (± 0.48)	1.53	0.217	0.01
PD	12.25 (± 0.30)	16.95 (± 0.38)	94.92	< 0.001	0.25
Negativity	2.25 (± 0.03)	2.36 (± 0.04)	5.51	0.020	0.02
VPT	− 0.05 (± 0.07)	0.08 (± 0.10)	1.01	0.316	< 0.01
ER_FAR_	− 0.11 (± 0.07)	0.05 (± 0.09)	2.10	0.148	< 0.01
Empathy_Aff_	21.88 (± 0.22)	21.26 (± 0.29)	3.01	0.084	0.01
INT_Acc_	0.50 (± 0.02)	0.422 (± 0.03)	3.96	0.048	0.01
IMI_Auto_	3.70 (± 3.00)	14.70 (± 3.87)	5.06	0.025	0.02

Values present means (± SE)

*AOF* Failure-related action orientation, *AOD* Demand-related action orientation, *PT* Perspective-taking, *EC* Empathic concern; *PD* Personal distress, *VPT* visual perspective taking, *ER*_*FAR*_ emotion regulation, *Empathy*_*Aff*_. affective empathy, *INT*_*Acc*_. interoceptive accuracy, *IMI*_*Auto*_. automatic imitation

Mediation analyses were then conducted on the four measures that did not differ significantly between the personality profiles: Empathic concern, visual perspective taking (VPT), emotion regulation, and affective empathy. Supplementary Table S4 presents the correlations between these four measures and all other dependent variables used to define potential mediators. Four models emerged that revealed variables serving as significant mediators in the influence of personality on measures of social cognition: (1) interoceptive accuracy fully mediated the effect on emotion regulation; (2) negativity and automatic imitation fully mediated the influence on affective empathy; (3) self-reported Perspective taking fully mediated the effect on VPT; (4) self-reported Personal distress, Perspective taking, and AOF partially mediated the influence on self-reported Empathic concern. The direction of these mediating influences is presented below, along with a description of the SEM results. It is important to stress that the term “full mediation” is a statistical concept—it does not imply that only the modelled variables underpin an observed relationship (see Hayes, 2018).

**Fig. 2:**
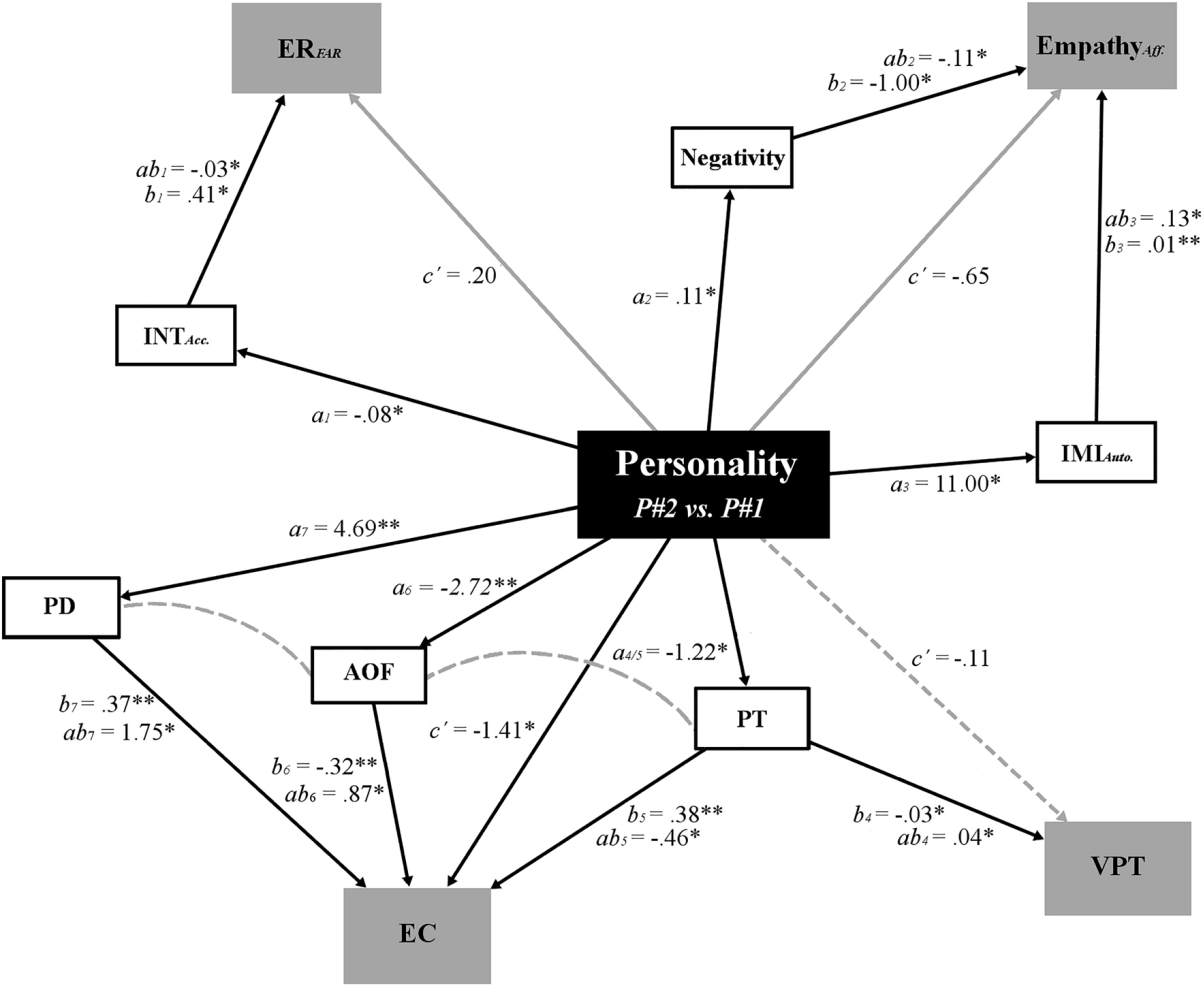
Unstandardised parameter estimates emerging from structural equation modelling (Hayes, 2013), expressing the values of profile 2 (P#2) relative to profile 1 (P#1). Grey nodes represent measures that did not differ significantly between personality profiles, while white nodes represent indirect pathways identified by mediation analyses. Dashed lines show changes to the mediation models suggested by modification indices: the curved lines represent covariances, and the straight line signifies a direct connection that was removed from the default model. *ER*_*FAR*_ emotion regulation, *Empathy*_*Aff*_. affective empathy, *INT*_*Acc*_. interoceptive accuracy, *IMI*_*Auto*_ automatic imitation, *PD* Personal distress, *PT* Perspective-taking, *EC* Empathic concern, *AOF* Failure-related action orientation, *VPT* visual perspective taking; *p < 0.05, **p < 0.01

When these separate mediation models were combined in an SEM analysis, modification indices suggested only three changes: the removal of a direct pathway from personality profile to VPT and two covariances—between AOF and self-reported Perspective taking, and AOF and Personal distress. The final model is presented in Fig. 2, which achieved excellent levels of fit with the observed data across discrepancy functions (*χ*^2^_[37]_ = 37.32, *p* = 0.45; root-mean-square error of approximation = 0.008) and comparative indices (normalised fit index = 0.896; comparative fit index = 0.999; Tucker Lewis index = 0.998). Moving clockwise through this structure from top left, the first branch presents an indirect pathway through which interoceptive accuracy fully mediates the influence of personality on emotion regulation. The *ab* path (indirect effect) reveals how the two personality profiles are estimated to differ on emotion regulation (the outcome) as a result of the effect of personality on interoceptive accuracy (the mediator), which, in turn, affects emotion regulation (the *b* path; cf. Hayes, 2013, pp. 92). In other words, while emotion regulation did not significantly differ *directly* between the two profiles, P#2 did have significantly poorer interoceptive accuracy and, through the positive relationship between interoception and emotion regulation, this was associated with a better emotion regulation (lower false-alarm rate).

The second branch encompasses a multimediator model, whereby there is no direct effect of personality on affective empathy but two indirect effects involving negativity and automatic imitation. First, individuals in P#2 exhibit stronger negativity than those in P#1, and this is associated inversely with affective empathy. As such, those in P#2 express a more negative affective state and, consequently, are less aroused by the emotions of others. P#2 also exhibit stronger automatic imitation relative to P#1, and automatic imitation is related positively with affective empathy. Due to the positive influence of automatic imitation on affective empathy, when taking into account the difference between profiles, people in P#2 show higher affective empathy than those in P#1. This inconsistent mediation reveals the opposing influences of negative affectivity and imitative tendencies on affective empathy, which has the potential to mask any difference between personality profiles.

The bottom right-hand branch comprises a single indirect pathway; those in P#2 report lower self-reported Perspective taking, and this is associated inversely with VPT. Keeping in mind that higher scores on VPT reflect poorer performance (inverted efficiency), the indirect path reveals that P#2 perform worse on the Director Task as a result of their self-reported lower Perspective taking.

The final branch presents another multimediator model, but this time comprising sub-scales from the self-report instruments. Relative to P#1, P#2 express lower Perspective taking, and through the positive relationship between Perspective taking and Empathic concern, in turn, demonstrate lower Empathic concern. Conversely, P#2 also exhibit higher Personal distress and, in turn, this is positively related to Empathic concern compared with P#1. Finally, people categorised as P#2 report lower AOF, with this associated negatively with scores of Empathic concern. Due to the negative influence of AOF on Empathic concern, P#2 express increased Empathic concern relative to P#1.

## Discussion

This study sought to elucidate the structure of inter-relationships and co-dependencies among discrete components of social cognition, and the personality mechanisms driving individual differences in this structure. From a large non-clinical sample, we acquired both performance-based and self-report measures of various socio-cognitive and -emotional capacities, and personality data with an instrument capable of delineating between personality style configurations. After profiling participants according to their personality style tendencies, we compared them on discrete facets of social cognition. Our results revealed three primary findings: (1) two dissociable personality profiles emerged that exhibited contrasting cognitive and affective dispositions; one showing flexibility among personality styles and adaptive action orientation, the other expressing persistence for certain styles and state orientation; (2) these profiles differed on a number of performance and self-report measures, revealing that mechanisms driving individual differences in personality also shape social cognition; (3) other facets of social cognition appear to mediate differences between these profiles in selected components, uncovering a hierarchical structure.

The two emerging profiles differed primarily in their preference for styles of Self-critical–Avoidant, Passive–Depressive, Spontaneous–Borderline, Optimistic–Rhapsodic, and Charming–Histrionic. According to the PSI theory, the lack of a strong preference across personality styles in Profile 1 (P#1) is indicative of greater flexibility and adaptation; individuals employing a larger repertoire of styles should be able to adjust to different situational demands more efficiently (Block, 2002; Kuhl, 2000a). In contrast, the strong tendency for Self-critical–Avoidant and Passive–Depressive styles shown by individuals in Profile 2 (P#2) suggests more inflexibility or rigidity. The styles preferred by P#2 indicate that they have a heightened sensitivity for negative affect together with a tendency to down-regulate positive affect. Interestingly, this replicates two previous findings: our group has identified a similar profile in an independent sample (*n* = 431; Czekóová, Shaw, & Urbánek, 2016), and a large-scale review of personality styles reports an “Overcontrolled prototype” that exhibits many of the same characteristics (Donnellan & Robbins, 2010). In line with these contrasting personality characteristics, individuals in P#2 expressed significantly stronger negativity than those in P#1. The PSI framework predicts that (a) chronic down-regulation of positive affect may induce state orientation, and (b) state-oriented individuals discriminate poorly between their own and others’ thoughts, wishes, and expectations (Koole et al., 2006; Kuhl, 1992). Consistent with the former premise, P#2 scored significantly lower on both Failure- and Demand-related action orientation (AOF and AOD, respectively), indicating a poorer ability to regulate their emotions when faced with negative events. This profile also scored lower on self-reported Perspective taking during social interactions, reported higher Personal distress when empathising with others, and exhibited much stronger imitative tendencies—all indices of inefficient self-other distinction (SOD).

The characteristics of these two personality profiles appear to resemble divergent patterns of cognitive control (e.g., Hommel & Colzato, 2017)—a fundamental capacity to override, restrain, or inhibit unwanted yet dominant action tendencies. It is proposed that individuals with high cognitive control can adapt more flexibly to highly demanding situations (*action orientation*) compared with individuals exhibiting inefficient cognitive control, who are less capable of activating appropriate adjustment processes (*state orientation*; Jostman & Koole, 2007). Consistent with this, prior research has shown that those with efficient cognitive control have a better emotion regulation and lower negative affect (Inzlicht et al., 2015). In this light, it could be argued that individuals in P#1 (higher flexibility) have, in general, a better cognitive control than those in P#2, enabling them to regulate their emotions more effectively and switch flexibly between cognitive self- and other-action/-affect representations. This would result in efficient SOD, leading to the reduced Personal distress when empathising and better control over imitative tendencies shown by P#1. In contrast, poorer cognitive control will lead to a cognitive system that is too inflexible to switch between alternative cognitive representations. Further research is needed to ascertain whether cognitive control does, indeed, underlie the two divergent personality profiles which we have observed. Furthermore, since considerable progress has been made in elucidating the various factors contributing to cognitive control (e.g., genetic and neurophysiological mechanisms; see Hommel, 2015), future research should investigate whether these same factors drive these two differential personality profiles.

Participants in P#2 reported significantly lower Perspective taking, and this was negatively associated with their VPT performance—with a lower tendency to take the perspective of others during social interaction, individuals are worse when resolving conflicts between their own and another’s perspective. This is, perhaps, unsurprising given the inefficient SOD of P#2, as indexed by their increased automatic imitation (Brass et al., 2009) and Personal distress (Lamm et al., 2016), and their apparent inefficiency in meta-control; avoiding ego-centric errors in perspective taking demands an ability to distinguish flexibly between competing self and other representations (Keysar et al., 2000; for a critical discussion, see Heyes, 2014; Santiesteban et al., 2012a). The state orientation of these individuals also suggests a bias in attentional focus towards the self, an additional factor shown to impact negatively on the ability to adopt another person’s viewpoint (Bukowski & Samson, 2016). Interestingly, individuals in P#2 showed a strong preference for Self-critical-Avoidant personality style, and patients diagnosed with Avoidant personality disorder present difficulties with metacognitive abilities of monitoring and decentration—the mislabelling of self-states and poor inference of others’ states, respectively (Moroni et al., 2016). This will result in both low ability and motivation to take the perspective of others, thereby reinforcing social withdrawal and hindering practice in perspective taking (for a related discussion, see Bird & Viding, 2014). In line with this, an association was reported recently between higher social anxiety and lower VPT (Pile et al., 2017). Studies have also shown that training individuals to withhold involuntary imitation enhances perspective-taking performance (Santiesteban et al., 2012b). It would be interesting, then, to see how individuals comprising P#2 respond to such SOD training; if effective, this simple intervention might prove beneficial for samples characterised by ego-centrism (Pile et al., 2017).

Given the significant difference in self-reported action orientation between profiles, it is surprising that personality did not influence emotion regulation directly; differences in false-alarm rate for affective stimuli on the emotional Go/No-Go task emerged only after interoceptive accuracy was included in the model. Moreover, the positive association between interoceptive accuracy and emotion regulation seems counter-intuitive; why should the greater interoceptive accuracy shown by individuals in P#1 lead to poorer emotion regulation? We suggest that this indicates a complex interaction between affective dispositions and the processing of physiological signals. While action-orientated individuals are more decisive and show less rumination under stress, when combined with enhanced processing of emotional stimuli facilitated by heightened interoception (see Critchley & Garfinkel, 2017), this might manifest as impulsivity on the emotional Go/No-Go task—that is, it may interfere with the withholding of responses to emotional expressions in others. At this point, it is important to stress that action orientation per se should not be considered superior to state orientation; individuals will benefit from a state-orientated approach in certain social situations, such as dangerous and unpredictable contexts (see Kuhl, 1992). Rather, effective emotion regulation requires the ability to switch flexibly between regulatory strategies in different situations. Likewise, it has been proposed that a balance between flexibility and persistence is needed for optimal cognitive control (Hommel, 2015).

Converging with the findings of Ainley et al. (2015), no association was observed between interoceptive accuracy and affective empathy, whether the latter is measured via the ratings of arousal in response to the emotions of others or by self-report instrument. Instead, our data reveal two mechanisms that together mediate the influence of personality mechanisms on empathic expression in opposing directions: First, negativity is related inversely with affective empathy, and through this pathway, individuals in P#2 exhibiting stronger negativity than P#1 also show *less* affective empathy. Negative affectivity has been shown to be associated positively with Personal distress (Eisenberg et al., 1994), and our data show that P#2 exhibited both stronger negativity and greater Personal distress than P#1. As such, this mediating effect of negativity might drive individuals in P#2 to withdraw, either implicitly or explicitly, from empathic engagement. Second, the strength of involuntary imitative tendencies is associated positively with affective empathy, and so greater imitative tendencies shown by P#2 may lead them to experience *more* arousal when empathising. Involuntary imitation is attributed to neural perception–action matching mechanisms (see Heyes, 2011); observing an action activates corresponding cortical motor representations, which serves to prime its execution. Through this mirroring process, we can simulate others’ actions implicitly (e.g., Gallese, 2013). In the same way, these mirroring mechanisms might permit the simulation of other emotions during empathy; the experience, observation, and imagination of an emotion engages overlapping brain responses (Jabbi, Bastiaansen, & Keysers, 2008; Wicker et al., 2003). Taking this into consideration, our data suggest that the ability to empathize *affectively* with others depends upon an interaction between our own current affective state and our tendency to simulate the emotional state of others. The difference which we observed in imitative tendencies between personality profiles stands in contrast to the findings of Butler et al. (2015) who observed no such influence of stable personality traits. This discrepancy could be due to methodological factors: first, imitation elicited by the action stimuli employed by Butler et al. (2015) is influenced heavily by spatial confounds (see Bertenthal et al., 2006; Shaw et al., 2017), but the present study used stimuli that avoided these effects and might have unmasked this relationship. Second, our person-centred approach to personality assessment might be more sensitive than the variable-centred approach adopted by Butler et al. (2015) in uncovering associations between this aspect of behaviour and personality.

The majority of sub-scales measured by the self-report instruments were clustered together in a single multimediator model. Lower Perspective taking, higher Personal distress, and reduced AOF reported by P#2 served as mediators in the influence of personality on Empathic concern. First, P#2 report higher Personal distress relative to P#1 and, in turn, this is associated positively with Empathic concern. Since the Empathic concern sub-scale is considered an explicit measure of affective empathy, this is entirely consistent with this group’s increased affective empathy. This may be indicative of greater self-other merging. Indeed, these same individuals exhibit greater automatic imitation, which suggests lower SOD. As discussed above, imitative tendencies are believed to reflect neural mirroring mechanisms through which the actions and emotions of others are understood through a process of implicit simulation. Inefficient distinction between self- and other-affect representations will lead to a misattribution of increased arousal levels experienced during this simulation process, leading to Personal distress when empathising (Lamm et al., 2016).

Conversely, P#2 report lower Perspective taking and AOF compared with P#1, and these two sub-scales are related to Empathic concern in an opposing manner; while reduced Perspective taking is associated with lower Empathic concern, decreased AOF is related to greater Empathic concern. Focusing on the first of these indirect pathways, the ego-centric attentional focus of this group seems to reduce the concern that they feel for others when empathising. The second pathway might be explained by the other inter-relationships and co-dependencies revealed in our structural model; P#2 report more state orientation during negative experiences and a greater tendency to imitate others, the latter of which leads to greater arousal during empathy. In the same way that imitation of others’ emotional states will lead to personal distress without compensatory SOD mechanisms, the state-orientated style of emotion regulation characterising this profile seems to result in greater empathic concern for the agent whose affect that they are simulating.

It is noteworthy that, aside from Perspective taking, none of the other sub-scales from self-report instruments served to mediate differences between personality profiles on the corresponding performance measures. This lack of association between explicit and implicit measures (see also Ainley et al., 2015; Böckler, Tusche, & Singer, 2016; Melchers et al., 2016) may suggest that they capture different facets of social cognition, or that our subjective experiences differ from our actual abilities. Our results indicate that many of the explicit measures are inter-related, and covariance between self-report measures suggest that individuals’ subjective view of themselves is consistent. As such, further research is needed to investigate whether self-report measures of social cognition differ from actual ability, and how personality might influence this.

## Conclusion

To our knowledge, this is the first investigation into social cognition that has collected data from a large sample on such an extensive battery of self-report and performance-based measures, and considered the influence of personality on patterns of inter-relationships and mediating effects among them. Our results reveal a hierarchical pattern of relationships among various components of social cognition, informing us about manner in which lower level cognitive mechanisms (e.g., visual perspective taking and self-other distinction) may influence higher level socio-emotional processes (e.g., affective empathy and emotion regulation). By moving beyond the modular approach that has dominated social cognition research to date, such findings provide a more detailed characterisation of individual differences in social behaviour. Using a person-centred approach to personality assessment, we have also shown that different configurations of personality systems exert an influence over discrete aspects of social cognition. At the most general level, these two profiles appear to reflect opposing capacities for cognitive control–flexibility versus rigidity/persistence—thereby providing a model for future research.

## Electronic supplementary material

Below is the link to the electronic supplementary material.

Supplementary material 1 (DOCX 5106 KB)

## References

[CR1] Ainley V, Maister L, Tsakiris M (2015). Heartfelt empathy? No association between interoceptive awareness, questionnaire measures of empathy, reading the mind in the eyes task or the director task. Frontiers in Psychology.

[CR2] Akaike H (1974). A new look at the statistical model identification. IEEE Transactions on Automatic Control.

[CR3] Baron RM, Kenny DA (1986). The moderator–mediator variable distinction in social psychological research: Conceptual, strategic, and statistical considerations. Journal of Personality and Social Psychology.

[CR4] Baron-Cohen S (2009). Autism: The empathizing-systemizing (E-S) theory. Annals of the New York Academy of Sciences.

[CR5] Bastiaansen JA, Thioux M, Keysers C (2009). Evidence for mirror systems in emotions. Philosophical Transactions of the Royal Society of London B: Biological Sciences.

[CR6] Baumann N, Kuhl J, Kazén M (2005). Left-hemispheric activation and self-infiltration: Testing a neuropsychological model of internalization. Motivation and Emotion.

[CR7] Bentley SV, Greenaway KH, Haslam A (2017). Cognition in context: Social inclusion attenuates the psychological boundary between self and other. Journal of Experimental Social Psychology.

[CR8] Bertenthal BI, Longo MR, Kosobud A (2006). Imitative response tendencies following observation of intransitive actions. Journal of Experimental Psychology: Human Perception and Performance.

[CR9] Bird G, Viding E (2014). The self to other model of empathy: providing a new framework for understanding empathy impairments in psychopathy, autism, and alexithymia. Neuroscience & Biobehavioral Reviews.

[CR10] Block J (2002). Personality as an affect-processing system: Toward an integrative theory.

[CR11] Böckler A, Tusche A, Singer T (2016). The structure of human prosociality: Differentiating altruistically motivated, norm motivated, strategically motivated, and self-reported prosocial behavior. Social Psychological and Personality Science.

[CR12] Brass M, Bekkering H, Prinz W (2001). Movement observation affects movement execution in a simple response task. Acta Psychologica.

[CR13] Brass M, Ruby P, Spengler S (2009). Inhibition of imitative behaviour and social cognition. Philosophical Transactions of the Royal Society of London B: Biological Sciences.

[CR14] Bruyer R, Brysbaert M (2011). Combining speed and accuracy in cognitive psychology: is the inverse efficiency score (IES) a better dependent variable than the mean reaction time (RT) and the percentage of errors (PE)?. Psychologica Belgica.

[CR15] Bukowski H, Samson D (2016). Can emotions influence level-1 visual perspective taking?. Cognitive Neuroscience.

[CR16] Butler EE, Ward R, Ramsey R (2015). Investigating the relationship between stable personality characteristics and automatic imitation. PLoS One.

[CR17] Catmur C, Walsh V, Heyes C (2009). Associative sequence learning: the role of experience in the development of imitation and the mirror system. Philosophical Transactions of the Royal Society of London B: Biological Sciences.

[CR18] Chartrand TL, Lakin JL (2013). The antecedents and consequences of human behavioral mimicry. Annual Review of Psychology.

[CR19] Cikara M, Bruneau E, Van Bavel JJ, Saxe R (2014). Their pain gives us pleasure: How intergroup dynamics shape empathic failures and counter-empathic responses. Journal of Experimental Social Psychology.

[CR20] Coll MP, Viding E, Rütgen M, Silani G, Lamm C, Catmur C, Bird G (2017). Are we really measuring empathy? Proposal for a new measurement framework. Neuroscience and Biobehavioral Reviews.

[CR21] Cracco E, Bardi L, Desmet C, Genschow O, Rigoni D, De Coster L, Brass M (2018). Automatic imitation: A meta-analysis. Psychological Bulletin.

[CR22] Critchley HD, Garfinkel SN (2017). Interoception and emotion. Current Opinion in Psychology.

[CR23] Critchley HD, Harrison NA (2013). Visceral influences on brain and behavior. Neuron.

[CR24] Critchley HD, Wiens S, Rotshtein P, Dolan RJ (2004). Neural systems supporting interoceptive awareness. Nature Neuroscience.

[CR25] Cronbach LJ, Furby L (1970). How we should measure” change”: Or should we?. Psychological bulletin.

[CR26] Czekóová, K., Pokorná, Z., & Špiláková, B. (2016). Vývoj metody pro posouzení sociální kognice. Sociální procesy a osobnost 2015—Otázky a výzvy, 67–72. Sborník příspěvků. Masarykova Univerzita Brno.

[CR27] Czekóová K, Shaw DJ, Urbánek T (2016). Personality systems, spirituality, and existential well-being: A person-centred perspective. Psychology of Religion and Spirituality.

[CR28] Davidson RJ, Putnam KM, Larson CL (2000). Dysfunction in the neural circuitry of emotion regulation—a possible prelude to violence. Science.

[CR29] Davis MH (1983). Measuring individual differences in empathy: Evidence for a multidimensional approach. Journal of Personality and Social Psychology.

[CR30] de Corte K, Buysse A, Verhofstadt L, Roeyers H, Ponnet K, Davis M (2007). Measuring empathic tendencies: Reliability and validity of the Dutch version of the interpersonal reactivity index. Psychologica Belgica.

[CR31] de Guzman M, Bird G, Banissy MJ, Catmur C (2016). Self–other control processes in social cognition: from imitation to empathy. Philosophical Transactions of the Royal Society of London B: Biological Sciences.

[CR32] de Vignemont F, Singer T (2006). The empathic brain: how, when and why?. Trends in Cognitive Sciences.

[CR33] Domschke K, Stevens S, Pfleiderer B, Gerlach AL (2010). Interoceptive sensitivity in anxiety and anxiety disorders: an overview and integration of neurobiological findings. Clinical Psychology Review.

[CR34] Donnellan MB, Robins RW (2010). Resilient, overcontrolled, and undercontrolled personality types: Issues and controversies. Social and Personality Psychology Compass.

[CR35] Dunn BD, Stefanovitch I, Evans D, Oliver C, Hawkins A, Dalgleish T (2010). Can you feel the beat? Interoceptive awareness is an interactive function of anxiety-and depression-specific symptom dimensions. Behaviour Research and Therapy.

[CR36] Dziobek I, Rogers K, Fleck S, Bahnemann M, Heekeren HR, Wolf OT, Convit A (2008). Dissociation of cognitive and emotional empathy in adults with Asperger syndrome using the Multifaceted Empathy Test (MET). Journal of Autism and Developmental Disorders.

[CR37] Eisenberg N, Fabes RA, Murphy B, Karbon M, Maszk P, Smith M, O’boyle C, Suh K (1994). The relations of emotionality and regulation to dispositional and situational empathy-related responding. Journal of Personality and Social Psychology.

[CR38] Epley N, Keysar B, van Boven L, Gilovich T (2004). Perspective taking as egocentric anchoring and adjustment. Journal of Personality and Social Psychology.

[CR39] Fertuck EA, Lenzenweger MF, Clarkin JF, Hoermann S, Stanley B (2006). Executive neurocognition, memory systems, and borderline personality disorder. Clinical Psychology Review.

[CR40] Filimon F, Nelson JD, Hagler DJ, Sereno MI (2007). Human cortical representations for reaching: mirror neurons for execution, observation, and imagery. Neuroimage.

[CR41] Frick C, Lang S, Kotchoubey B, Sieswerda S, Dinu-Biringer R, Berger M, Veser S, Essig M, x`& Barnow S (2012). Hypersensitivity in borderline personality disorder during mindreading. PLoS One.

[CR42] Frith CD, Frith U (2012). Mechanisms of social cognition. Annual Review of Psychology.

[CR43] Füstös J, Gramann K, Herbert BM, Pollatos O (2012). On the embodiment of emotion regulation: Interoceptive awareness facilitates reappraisal. Social Cognitive and Affective Neuroscience.

[CR44] Gallese V (2013). Mirror neurons, embodied simulation and a second-person approach to mindreading. Cortex.

[CR45] Garfinkel SN, Seth AK, Barrett AB, Suzuki K, Critchley HD (2015). Knowing your own heart: Distinguishing interoceptive accuracy from interoceptive awareness. Biological Psychology.

[CR46] Genschow O, van Den Bossche S, Cracco E, Bardi L, Rigoni D, Brass M (2017). Mimicry and automatic imitation are not correlated. PLoS One.

[CR47] Gignac GE, Szodorai ET (2016). Effect size guidelines for individual differences researchers. Personality and Individual Differences.

[CR48] Gilet AL, Mella N, Studer J, Grühn D, Labouvie-Vief G (2013). Assessing dispositional empathy in adults: A French validation of the interpersonal reactivity index (IRI). Canadian Journal of Behavioural Science.

[CR49] Grezes J, Decety J (2001). Functional anatomy of execution, mental simulation, observation, and verb generation of actions: A meta-analysis. Human Brain Mapping.

[CR50] Gross JJ (2002). Emotion regulation: Affective, cognitive, and social consequences. Psychophysiology.

[CR51] Grynberg D, Pollatos O (2015). Perceiving one’s body shapes empathy. Physiology & Behavior.

[CR52] Happé F, Cook JL, Bird G (2017). The structure of social cognition: In(ter) dependence of sociocognitive processes. Annual Review of Psychology.

[CR53] Hawk ST, Keijsers L, Branje SJ, Graaff JVD, Wied MD, Meeus W (2013). Examining the interpersonal reactivity index (IRI) among early and late adolescents and their mothers. Journal of Personality Assessment.

[CR54] Hayes AF (2013). Introduction to mediation, moderation, and conditional process analysis: A regression-based approach.

[CR55] Hayes AF (2018). Introduction to mediation, moderation, and conditional process analysis: A regression-based approach.

[CR56] Hengartner MP, Müller M, Rodgers S, Rössler W, Ajdacic-Gross V (2014). Interpersonal functioning deficits in association with DSM-IV personality disorder dimensions. Social Psychiatry and Psychiatric Epidemiology.

[CR57] Herbert BM, Muth ER, Pollatos O, Herbert C (2012). Interoception across modalities: On the relationship between cardiac awareness and the sensitivity for gastric functions. PLoS One.

[CR58] Herpertz SC, Bertsch K (2014). The social-cognitive basis of personality disorders. Current Opinion in Psychiatry.

[CR59] Heyes C (2011). Automatic imitation. Psychological Bulletin.

[CR60] Heyes C (2014). Submentalizing: I am not really reading your mind. Perspectives on Psychological Science.

[CR61] Hogeveen J, Obhi SS (2013). Automatic imitation is automatic, but less so for narcissists. Experimental Brain Research.

[CR62] Hommel B (2015). Between persistence and flexibility: The Yin and Yang of action control. Advances in Motivation Science.

[CR63] Hommel B, Colzato LS (2017). The social transmission of metacontrol policies: Mechanisms underlying the interpersonal transfer of persistence and flexibility. Neuroscience and Biobehavioral Reviews.

[CR64] Iacoboni M (2009). Imitation, empathy, and mirror neurons. Annual Review of Psychology.

[CR65] Inzlicht M, Bartholow BD, Hirsh JB (2015). Emotional foundations of cognitive control. Trends in Cognitive Sciences.

[CR66] Jabbi M, Bastiaansen J, Keysers C (2008). A common anterior insula representation of disgust observation, experience and imagination shows divergent functional connectivity pathways. PLoS One.

[CR67] Jostmann NB, Koole SL (2007). On the regulation of cognitive control: Action orientation moderates the impact of high demands in stroop interference tasks. Journal of Experimental Psychology: General.

[CR68] Kanske P, Böckler A, Singer T (2015). Models, mechanisms and moderators dissociating empathy and theory of mind. Current Topics in Behavioural Neuroscience.

[CR69] Kazén M, Kuhl J, Quirin M (2015). Personality interacts with implicit affect to predict performance in analytic versus holistic processing. Journal of Personality.

[CR70] Kenny DA, Judd CM (2014). Power anomalies in testing mediation. Psychological Science.

[CR71] Keysar B, Barr DJ, Balin JA, Brauner JS (2000). Taking perspective in conversation: The role of mutual knowledge in comprehension. Psychological Science.

[CR72] Kline, R. B. (2015). *Principles and practice of structural equation modeling*. New York, London: Guilford publications.

[CR73] Knoll JF, Hodapp V (1992). A comparison between two methods for assessing heartbeat perception. Psychophysiology.

[CR74] Knowles ML (2012). Social rejection increases perspective taking. Journal of Experimental Social Psychology.

[CR75] Konstantareas MM, Stewart K (2006). Affect regulation and temperament in children with autism spectrum disorder. Journal of Autism and Developmental Disorders.

[CR76] Koole SL, Fockenberg DA (2011). Implicit emotion regulation under demanding conditions: The moderating role of action versus state orientation. Cognition and Emotion.

[CR77] Koole SL, Kuhl J, Jostmann NB, Finkenauer C, Vohs KD, Finkel EJ (2006). Self-regulation in interpersonal relationships: the case of action versus state orientation. Self and relationships: Connecting intrapersonal and interpersonal processes.

[CR78] Ku G, Wang CS, Galinsky AD (2010). Perception through a perspective-taking lens: Differential effects on judgment and behaviour. Journal of Experimental Social Psychology.

[CR79] Kuhl J (1992). A theory of self-regulation: Action versus state orientation, self-discrimination, and some applications. Applied Psychology.

[CR80] Kuhl J, Kuhl J, Beckmann J (1994). Action versus state orientation: Psychometric properties of the Action Control Scale (ACS-90). Volition and personality: Action versus state orientation.

[CR81] Kuhl J (2000). A theory of self-development: Affective fixation and the STAR model of personality disorders and related styles. Advances in Psychology.

[CR82] Kuhl J (2000). The volitional basis of personality systems interaction theory: Applications in learning and treatment contexts. International Journal of Educational Research.

[CR83] Kuhl J, Kazén M (2002). PSSI—Inventář stylů osobnosti a poruch osobnosti.

[CR84] Kuhl J, Kazén M, Koole SL (2006). Putting self-regulation theory into practice: A user's manual. Applied Psychology.

[CR85] Lamm C, Bukowski H, Silani G (2016). From shared to distinct self–other representations in empathy: Evidence from neurotypical function and socio-cognitive disorders. Philosophical Transactions of the Royal Society: B.

[CR86] Lamm C, Decety J, Singer T (2011). Meta-analytic evidence for common and distinct neural networks associated with directly experienced pain and empathy for pain. Neuroimage.

[CR87] Langner O, Dotsch R, Bijlstra G, Wigboldus DH, Hawk ST, Van Knippenberg AD (2010). Presentation and validation of the Radboud Faces Database. Cognition and Emotion.

[CR88] Lazarsfeld PF, Henry NW (1968). Latent structure analysis.

[CR89] Leighton J, Bird G, Orsini C, Heyes C (2010). Social attitudes modulate automatic imitation. Journal of Experimental Social Psychology.

[CR90] Lo Y, Mendell N, Rubin D (2001). Testing the number of components in a normal mixture. Biometrika.

[CR91] Mazza M, Pino MC, Mariano M, Tempesta D, Ferrara M, De Berardis D, Valenti M (2014). Affective and cognitive empathy in adolescents with autism spectrum disorder. Frontiers in Human Neuroscience.

[CR92] McLachlan G, Peel D (2000). Finite mixture models.

[CR93] Melchers MC, Li M, Haas BW, Reuter M, Bischoff L, Montag C (2016). Similar personality patterns are associated with empathy in four different countries. Frontiers in Psychology.

[CR94] Mogan R, Fischer R, Bulbulia JA (2017). To be in synchrony or not? A meta-analysis of synchrony’s effects on behavior, perception, cognition and affect. Journal of Experimental Social Psychology.

[CR95] Moore RC, Dev SI, Jeste DV, Dziobek I, Eyler LT (2015). Distinct neural correlates of emotional and cognitive empathy in older adults. Psychiatry Research: Neuroimaging.

[CR96] Morgan GB (2015). Mixed mode latent class analysis: An examination of fit index performance for classification. Structural Equation Modeling: A Multidisciplinary Journal.

[CR97] Moroni F, Procacci M, Pellecchia G, Semerari A, Nicolò G, Carcione A, Pedone R, Colle L (2016). Mindreading dysfunction in avoidant personality disorder compared with other personality disorders. The Journal of Nervous and Mental Disease.

[CR98] Muthén, L. K., & Muthén, B. O. (1998–2012). *Mplus user’s guide* (7th ed.). Los Angeles: Muthén & Muthén.

[CR99] New AS, Rot MAH, Ripoll LH, Perez-Rodriguez MM, Lazarus S, Zipursky E, Weinstein SR, Koenigsberg HW, Hazlett EA, Goodman M, Siever LJ (2012). Empathy and alexithymia in borderline personality disorder: Clinical and laboratory measures. Journal of Personality Disorders.

[CR100] Nylund KL, Asparouhov T, Muthén BO (2007). Deciding on the number of classes in latent class analysis and growth mixture modeling: A Monte Carlo simulation study. Structural Equation Modeling.

[CR101] Obhi SS, Hogeveen J, Giacomin M, Jordan CH (2014). Automatic imitation is reduced in narcissists. Journal of Experimental Psychology: Human Perception and Performance.

[CR102] Okun MA, Shepard SA, Eisenberg N (2000). The relations of emotionality and regulation to dispositional empathy-related responding among volunteers-in-training. Personality and Individual Differences.

[CR103] Paulus MP, Stein MB (2010). Interoception in anxiety and depression. Brain Structure and Function.

[CR104] Peterson JL, Bellows A, Peterson S (2015). Promoting connection: Perspective-taking improves relationship closeness and perceived regard in participants with low implicit self-esteem. Journal of Experimental Social Psychology.

[CR105] Pile V, Haller SP, Hiu CF, Lau JY (2017). Young people with higher social anxiety are less likely to adopt the perspective of another: Data from the director task. Journal of Behavior Therapy and Experimental Psychiatry.

[CR106] Quirin M, Bode RC (2014). An alternative to self-reports of trait and state affect. European Journal of Psychological Assessment.

[CR107] Quirin M, Bode RC, Kuhl J (2011). Recovering from negative events by boosting implicit positive affect. Cognition and Emotion.

[CR108] Quirin M, Kazén M, Kuhl J (2009). When nonsense sounds happy or helpless: The implicit positive and negative affect test (IPANAT). Journal of Personality and Social Psychology.

[CR109] Ramaswamy V, DeSarbo WS, Reibstein DJ, Robinson WT (1993). An empirical pooling approach for estimating marketing mix elasticities with PIMS data. Marketing Science.

[CR110] Rogers K, Dziobek I, Hassenstab J, Wolf OT, Convit A (2007). Who cares? Revisiting empathy in asperger syndrome. Journal of Autism and Developmental Disorders.

[CR111] Samson D, Apperly IA, Braithwaite JJ, Andrews BJ, Bodley Scott SE (2010). Seeing it their way: Evidence for rapid and involuntary computation of what other people see. Journal of Experimental Psychology. Human Perception and Performance.

[CR112] Santiesteban I, Banissy MJ, Catmur C, Bird G (2012). Enhancing social ability by stimulating right temporoparietal junction. Current Biology.

[CR113] Santiesteban I, White S, Cook J, Gilbert SJ, Heyes C, Bird G (2012). Training social cognition: From imitation to theory of mind. Cognition.

[CR114] Schandry R (1981). Heart beat perception and emotional experience. Psychophysiology.

[CR115] Schipper M, Petermann F (2013). Relating empathy and emotion regulation: Do deficits in empathy trigger emotion dysregulation?. Social Neuroscience.

[CR116] Schwarz G (1978). Estimating the dimension of a model. Annals of Statistics.

[CR117] Semerari A, Colle L, Pellecchia G, Buccione I, Carcione A, Dimaggio G, Nicolò G, Procacci M, Pedone R (2014). Metacognitive dysfunctions in personality disorders: Correlations with disorder severity and personality styles. Journal of Personality Disorders.

[CR118] Semerari A, Colle L, Pellecchia G, Carcione A, Conti L, Fiore D, Moroni F, Nicolò G, Procacci M, Pedone R (2015). Personality disorders and mindreading: Specific impairments in patients with borderline personality disorder compared to other PDs. The Journal of Nervous and Mental Disease.

[CR119] Shamay-Tsoory SG (2011). The neural bases for empathy. The Neuroscientist.

[CR120] Shamay-Tsoory SG, Aharon-Peretz J, Perry D (2009). Two systems for empathy: a double dissociation between emotional and cognitive empathy in inferior frontal gyrus versus ventromedial prefrontal lesions. Brain.

[CR121] Shaw DJ, Czekóová K, Porubanová M (2017). Orthogonal-compatibility effects confound automatic imitation: Implications for measuring self–other distinction. Psychological Research Psychologische Forschung.

[CR122] Silani G, Lamm C, Ruff CC, Singer T (2013). Right supramarginal gyrus is crucial to overcome emotional egocentricity bias in social judgments. Journal of Neuroscience.

[CR123] Spengler S, Bird G, Brass M (2010). Hyperimitation of actions is related to reduced understanding of others’ minds in autism spectrum conditions. Biological Psychiatry.

[CR124] Steinbeis N (2016). The role of self–other distinction in understanding others’ mental and emotional states: neurocognitive mechanisms in children and adults. Philosophical Transactions of the Royal Society: B.

[CR125] Su HJ, Lee S, Ding J, Comer LB (2005). Relations among measures of trait empathy, empathetic response, and willingness to get involved in customer-contact situations. Psychological Reports.

[CR126] Tabachnik BG, Fidell SL (2013). Multicollinearity and singularity. Using multivariate statistics.

[CR127] Thoma P, Friedmann C, Suchan B (2013). Empathy and social problem solving in alcohol dependence, mood disorders and selected personality disorders. Neuroscience & Biobehavioral Reviews.

[CR128] Tomova L, von Dawans B, Heinrichs M, Silani G, Lamm C (2014). Is stress affecting our ability to tune into others? Evidence for gender differences in the effects of stress on self–other distinction. Psychoneuroendocrinology.

[CR129] Tottenham N, Hare TA, Casey BJ (2011). Behavioral assessment of emotion discrimination, emotion regulation, and cognitive control in childhood, adolescence, and adulthood. Frontiers in Psychology.

[CR130] Tsakiris M, Critchley H (2016). Interoception beyond homeostasis: Affect, cognition and mental health. Philosophical Transactions of the Royal Society B: Biological Sciences.

[CR131] Urbánek T, Marček V (2016). Investigating the effectiveness of working memory training in the context of Personality Systems Interaction theory. Psychological Research Psychologische Forschung.

[CR132] van der Ploeg MM, Brosschot JF, Thayer JF, Verkuil B (2016). The implicit positive and negative affect test: Validity and relationship with cardiovascular stress-responses. Frontiers in Psychology.

[CR133] Weafer J, Baggott MJ, de Wit H (2013). Test–retest reliability of behavioral measures of impulsive choice, impulsive action, and inattention. Experimental and Clinical Psychopharmacology.

[CR134] Werner NS, Kerschreiter R, Kindermann NK, Duschek S (2013). Interoceptive awareness as a moderator of affective responses to social exclusion. Journal of Psychophysiology.

[CR135] Wicker B, Keysers C, Plailly J, Royet JP, Gallese V, Rizzolatti G (2003). Both of us disgusted in my insula: The common neural basis of seeing and feeling disgust. Neuron.

[CR136] Zhao X, Lynch JG, Chen Q (2010). Reconsidering Baron and Kenny: Myths and truths about mediation analysis. Journal of Consumer Research.

