# Supplementary material for: You ≠ me: individual differences in the structure of social cognition
Source: Psychol Res. 2018 Oct 15;84(4):1139–56. doi: 10.1007/s00426-018-1107-3 (PMC7239802; doi:10.1007/s00426-018-1107-3)
Supplement: Supplementary file 1 — Supplementary material 1 (DOCX 5106 KB) [file 426_2018_1107_MOESM1_ESM.docx]

Supplementary Table S1. Basic demographics.

|  |  | Combined | P#1 | P#2 | *p* Value |
| --- | --- | --- | --- | --- | --- |
| Gender | Male | 117 | 77 | 40 | .326 |
|  | Female | 173 | 104 | 69 |  |
|  | Age | 23.0 (3.2) | 23.2 (3.2) | 22.8 (3.1) | .369 |
| Education | High school | 179 | 110 | 69 | .685 |
|  | Bachelor | 3 | 1 | 2 |  |
|  | Master | 75 | 48 | 27 |  |
|  | Doctorate | 33 | 22 | 11 |  |
| Specialisation | Humanities | 116 | 74 | 42 | .646 |
|  | Science | 79 | 48 | 31 |  |
|  | Technical | 34 | 23 | 11 |  |
|  | Agricultural | 8 | 6 | 2 |  |
|  | Arts | 8 | 3 | 5 |  |
|  | Other | 45 | 27 | 18 |  |
| Marital status | Single | 205 | 126 | 79 | .200 |
|  | Partner | 75 | 51 | 24 |  |
|  | Married | 10 | 4 | 6 |  |
| Occupation | Student | 251 | 152 | 99 | .060 |
|  | Employed/Self-employed | 34 | 27 | 7 |  |
|  | At home/parental leave | 5 | 2 | 3 |  |

*Note*. Values present means (±SE) for the final sample (*N* = 290). P-values present the significance of chi-square or t-test comparisons between profiles.

Supplementary Table S2. Internal consistency for sub-scales of self-report instruments.

| Instrument | Sub-scale/Dimension | Cronbach’s *α* |
| --- | --- | --- |
| ACS | AOF | .76 |
|  | AOD | .74 |
| IRI | PT | .74 |
|  | EC | .79 |
|  | PD | .73 |
| PSDI | Assertive–Antisocial | .81 |
|  | Wilful–Paranoid | .81 |
|  | Reserved–Schizoid | .83 |
|  | Self-critical–Avoidant | .81 |
|  | Conscientious–Compulsive | .79 |
|  | Intuitive–Schizotypal | .86 |
|  | Optimistic–Rhapsodic | .83 |
|  | Ambitious–Narcissistic | .64 |
|  | Critical–Negativistic | .67 |
|  | Loyal–Dependent | .79 |
|  | Spontaneous–Borderline | .76 |
|  | Charming–Histrionic | .86 |
|  | Passive–Depressive | .80 |
|  | Unselfish–Self-sacrificing | .76 |

*Note*: *Abbreviations:* AOF = Failure-related action orientation, AOD = Demand-related action orientation, PT = Perspective-taking, EC = Empathic concern, PD = Personal distress.

Supplementary Table S3. Fit indices for different solutions emerging from Latent Profile Analysis.

| **Models** | **No. Profiles in Solution** | | | | |
| --- | --- | --- | --- | --- | --- |
|  | **1** | **2** | **3** | **4** | **5** |
| *No. parameters* | 28 | 43 | 58 | 73 | 88 |
| *Log Likelihood* | -12936 | -12610 | -12485 | -12383 | -12308 |
| *AIC* | 25929 | 25305 | 25085 | 24912 | 24792 |
| *BIC* | 26033 | 25465 | 25301 | 25183 | 25119 |
| *Adjusted BIC* | 25944 | 25329 | 25117 | 24952 | 24840 |
| *Entropy* | - | .89 | .88 | .89 | .90 |
| *LMR LRT (p)* | - | .000 | .203 | .295 | .063 |
| *Bootstrap LRT (p)* | - | < .001 | < .001 | < .001 | < .001 |
| *No. participants/profile* | 303 | 187  116 | 160  55  88 | 130  54  35  84 | 132  31  47  59  34 |

*Note:* AIC = Akaike Information Criteria, BIC = Bayesian Information Criterion, LRT = Likelihood Ratio Test, LMR = Lo-Mendell-Rubin.

Supplementary Table S4. Correlations among all measures of social cognition.

|  | AOF | AOD | PT | EC | PD | Negativity | VPT | ER*_FAR_* | Empathy*_Aff._* | INT*_Acc._* | IMI*_Auto._* |
| --- | --- | --- | --- | --- | --- | --- | --- | --- | --- | --- | --- |
| AOF | 1 |  |  |  |  |  |  |  |  |  |  |
| AOD | .29^**^ | 1 |  |  |  |  |  |  |  |  |  |
| PT | .22^**^ | .18^**^ | 1 |  |  |  |  |  |  |  |  |
| EC | -.23^**^ | .02 | .28^**^ | 1 |  |  |  |  |  |  |  |
| PD | -.50^**^ | -.31^**^ | -.12^*^ | .33^**^ | 1 |  |  |  |  |  |  |
| Negativity | >.01 | -.03 | -.05 | -.02 | -.03 | 1 |  |  |  |  |  |
| VPT | -.04 | -.03 | -.15^*^ | -.07 | -.04 | .14^*^ | 1 |  |  |  |  |
| ER*_FAR_* | -.07 | -.05 | -.02 | .02 | .06 | -.002 | -.04 | 1 |  |  |  |
| Empathy*_Aff._* | .04 | -.01 | .05 | .02 | -.03 | -.13^*^ | -.01 | -.04 | 1 |  |  |
| INT*_Acc._* | .14^*^ | .12^*^ | .10 | -.05 | -.01 | -.01 | -.05 | .13^*^ | .01 | 1 |  |
| IMI*_Auto._* | -.13^*^ | -.05 | .01 | -.04 | .06 | .04 | .12^*^ | -.07 | .14^*^ | -.10 | 1 |
| *Note*: *Abbreviations:* AOF = Failure-related action orientation, AOD = Demand-related action orientation, PT = Perspective-taking, EC = Empathic concern, PD = Personal distress, VPT = Visual perspective taking, ER*_FAR_* = Emotion regulation, Empathy*_Aff._* = Affective empathy, INT*_Acc._* = Interoceptive accuracy, IMI*_Auto._* = Automatic imitation. Values represent Pearson correlation coefficients across the final sample (*N* = 290). * = *p* < .05; ** = *p* < .01. | | | | | | | | | | |  |

Experimental stimuli.


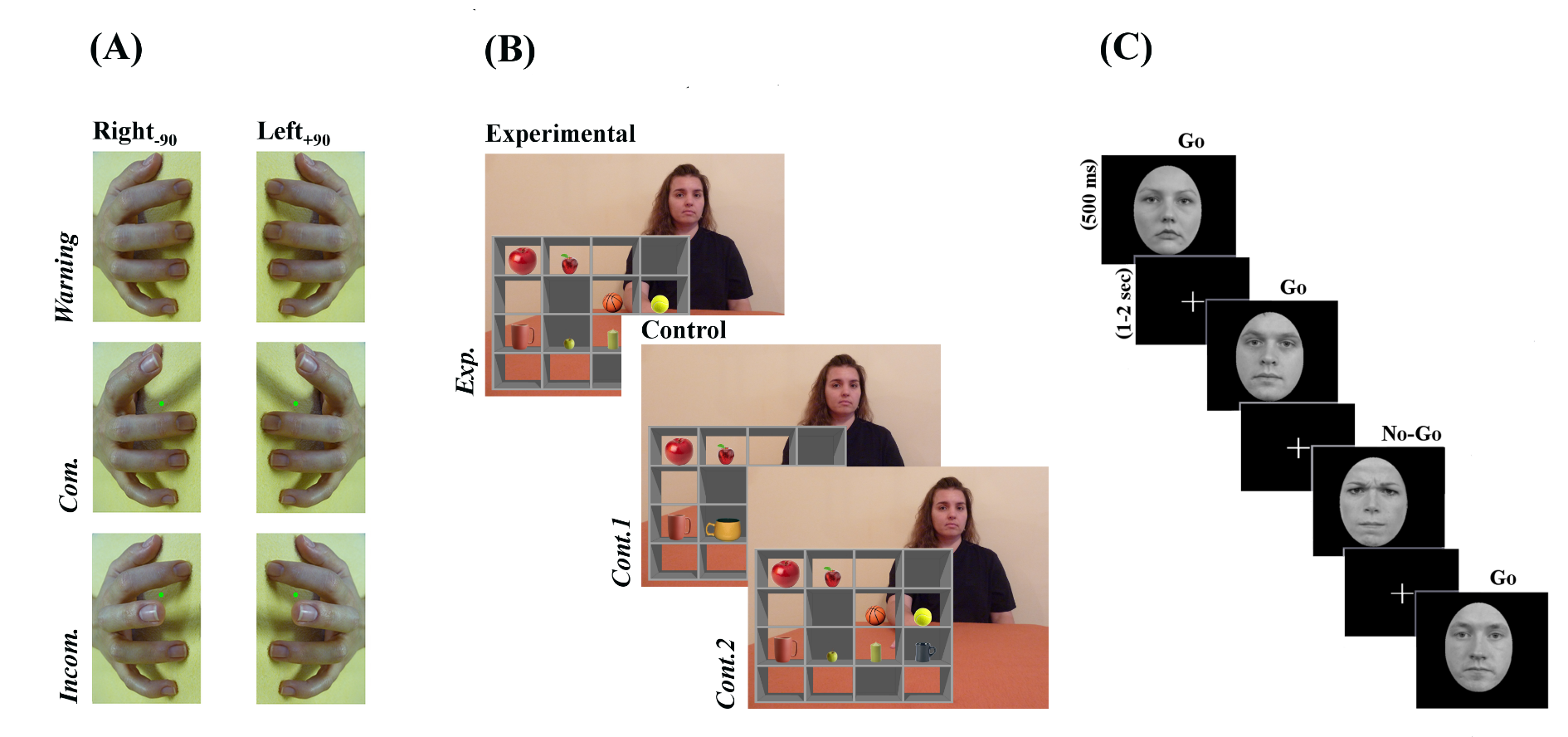


*Supplementary Figure S1*: Example stimuli used in the two blocks of the stimulus-response compatibility task (*A*); the three conditions of the Director Task (*B*); and a segment of a neutral-angry block from the emotional Go/No-Go task, in which the angry expression was the No-Go stimulus (*C*).

*
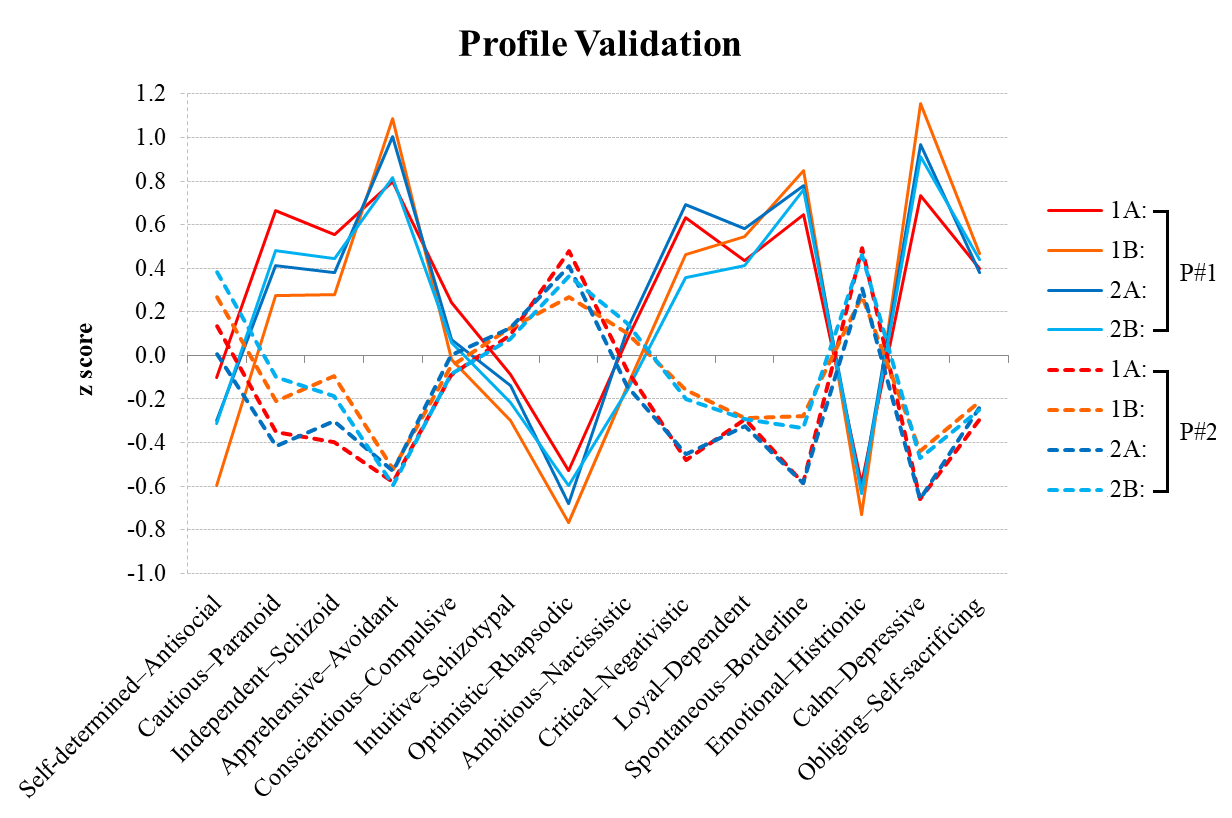
*

*Supplementary Figure S2*: Results of the split-half validation process. Strikingly similar profiles emerged from independent two-class latent profile analyses applied to each of the four subsets of participants from two randomised half-splits of the sample. *N* for each independent LPA: 1A = 158, 1B = 145, 2A = 167, 2B = 136.
